# The quality of life experienced by persons with spinal cord injury in Finland – a mixed methods study

**DOI:** 10.3389/fresc.2026.1829111

**Published:** 2026-10-21

**Authors:** Kaarina Eskola, Susanna Tallqvist, Minna Zechner, Jari Arokoski, Eerika Koskinen, Marketta Rajavaara, Sinikka Hiekkala

**Affiliations:** 1 Doctoral Programme in Social Sciences, University of Helsinki, Helsinki, Finland; 2 Intensive Rehabilitation Unit, Tampere University Hospital, Tampere, Finland; 3 Arcada University of Applied Sciences, Helsinki, Finland; 4 Faculty of Social Sciences, University of Helsinki, Helsinki, Finland; 5 Spinal Cord Injury Outpatient Clinic, Division of Rehabilitation, Department of Internal Medicine and Rehabilitation, Helsinki University Hospital and University of Helsinki, Helsinki, Finland; 6 Information Production and Foresight, The Finnish Association of People with Physical Disabilities, Helsinki, Finland; 7 Faculty of Sport and Health Sciences, University of Jyväskylä, Jyväskylä, Finland

**Keywords:** mixed-method research, psychosocial adaptation, quality of life, rehabilitation, self-efficacy, spinal cord injury

## Abstract

**Introduction:**

Spinal cord injury (SCI) affects an individual's quality of life (QOL) by impairing body functions and structures, which in turn lead to reduced functional capacity and participation restrictions. These outcomes are further shaped by environmental factors. The objective of this mixed-method study was to investigate the factors associated with QOL among persons with SCI in Finland.

**Methods:**

A convergent parallel mixed methods design with quantitative and qualitative data was used. Quantitative data were obtained from 879 individuals with SCI participating in the first nationwide community survey for the SCI population in Finland (FinSCI 2019). QOL was assessed using the PROMIS® Global Health survey question (Global02), and associations with sociodemographic and injury-related variables were analyzed using multivariable ordinal regression. Qualitative data were collected through interviews with 45 volunteer survey respondents and analyzed using inductive content analysis. Quantitative and qualitative data generated were then triangulated to develop a more complete interpretation of the QOL experienced by SCI population and integrated results were assessed for concordance, discordance or expansion during the discussion stage of this report.

**Results:**

Impaired overall health and poor management of secondary health conditions (SHCs) were associated with reduced functional ability and poorer QOL, whereas SCI severity was not independently associated with QOL in the adjusted quantitative model. Factors associated with improved QOL included immediate social support, high self-esteem, psychosocial adaptation, and personal growth over time. Conversely, older age, secondary health problems, and inadequate services were linked to poorer QOL. High self-efficacy and the ability to function within personal limitations while maintaining autonomy were associated with good QOL, whereas poor QOL was characterized by reduced activity, constrained daily life, and lack of spontaneity.

**Discussion:**

The findings highlight the complex interplay between health status, functional ability, psychosocial resources, and the adequacy of servicesin shaping QOL among people with SCI in Finland, whereas injury severity itself was not independently associated with QOL. Addressing these multidimensional factors is essential in rehabilitation and long-term follow-up to support well-being and participation in everyday life.

## 1 Introduction

Spinal cord injury (SCI) has a multidimensional impact on an individual's quality of life (QOL), as it affects body structures and functions, often resulting in impairments in functional abilities, limitations in daily activities, and restrictions in participation, all of which are further influenced by environmental factors (1–3).

Multiple studies have found that SCI negatively affects QOL, well-being, and average life satisfaction (2, 4–9). However, these diverse and evolving interpretations of the QOL construct have been shown to change over time following the injury due to the individualistic nature of the concept of QOL (10). In the previous literature, factors that are seen to promote QOL among individuals with SCI include having meaningful relationships, taking responsibility for one's own life and having the opportunity to make decisions about it, as well as being able to engage in activities that are personally meaningful (11), whereas factors found to diminish QOL include pain, spasticity, incontinence, less physical independence and mobility, pressure sores and dysreflexia, inadequate income, boredom, dissatisfaction with occupations and perceptions of having reduced control over life (11–13).

A substantial body of research has been conducted on the QOL of individuals with SCI, employing a range of methodologies and approaches. Many studies have analyzed the QOL of individuals with SCI using either purely quantitative methods (14) or qualitative methods (11). Despite the availability of various tools for assessing QOL, there is no universally accepted definition of QOL within studies on persons with SCI (9, 11, 14, 15), which makes cross-study comparisons challenging.

Additionally, the focus on quantitative methods, often at the cost of qualitative approaches, limits a full and detailed understanding of this complex concept (14). A thorough understanding of the subjective experience of QOL requires research methodologies that can examine both the content and the contextual dimensions of an individual's life (14).

QOL is a dynamic, multidimensional and context-dependent phenomenon, which cannot always be accurately or adequately captured through standardized measurement tools (10, 16). The findings from previous studies suggest that QOL should be measured using subjective indicators, if not entirely, at least in combination with objective measures, in order to identify what truly matters to individuals with SCI (17).

QOL has various definitions. As a concept, the World Health Organization (WHO) defines QOL as an individual's perception of their position in life in the context of the culture and value systems in which they live and in relation to their goals, expectations, standards and concerns (18). Life satisfaction has also been used in studies of people with SCI, which is commonly referred to as an individual's subjective judgment upon the current life situation in relation to their own standards and expectations and reflections the perception to which degree aspirations and achievements (19). Subjective well-being has also been defined as synonymous with QOL and life satisfaction (12). In her systematic review, Whalley Hammell (11) conceptualizes QOL as “the experience of a life worth living”.

In Finland, the acute care, subacute rehabilitation, and life-long follow-up of patients with SCI were centralized in three university hospitals in 2011 (20), enabling more efficient data collection on the SCI population. Since then, the incidence of SCIs in Finland has been investigated in two studies: one reported an annual incidence of traumatic SCI (TSCI) at 38 per million (21), while the other found the incidence of non-traumatic SCI (NTSCI) to be 54.1 per million per year (22). The NTSCI patients sustained their injuries due to degenerative diseases, whereas the primary cause of injury among TSCI patients was low-level falls. One aim of the FinSCI study was to gain a comprehensive understanding of the life situation of people living with SCI in Finland. Therefore, the aim of current study was to describe the subjective QOL of persons with SCI in Finland by using a multi-method research approach, in order to obtain a comprehensive picture of the self-rated QOL and the factors associated with it. In this study, QOL refers to the physical, psychological, and social dimensions of well-being (16) as subjectively expressed by individuals with SCI, which may be influenced by illness or injury itself as well as contextual factors.

## 2 Material and methods

### 2.1 Design

A mixed-method approach using questionnaire and semi-structured interviews was chosen to analyze the outcomes of QOL experienced by SCI population in Finland. The participants for the study were collected from the data of the first nationwide community survey for this population, the Finnish Spinal Cord Injury Study (FinSCI), in which the study subjects were recruited by exploring the patient registers in three university hospitals (Oulu, Tampere and Helsinki) responsible for acute care, rehabilitation and lifelong follow-up of persons with SCI in Finland (23). The purpose of FinSCI was to identify factors related to the health and functioning of people with SCI, their challenges with accessibility, and how such factors are interconnected. One aim of the FinSCI was to reinforce and complement the data produced by establishing a dialogue among various types of data. The inclusion criteria for FinSCI were at least 16 years of age, permanently living in Finland, non-traumatic or traumatic SCI classified with an ASIA Impairment Scale (AIS) grade of A, B, C or D, and persons were patients at three SCI outpatient clinics responsible for lifelong follow-up care in Finland. The protocol of the FinSCI is presented elsewhere (23), and it was approved by the Hospital District of Helsinki and Uusimaa (HUS) Coordinating Ethics Committee (HUS/1776/2017).

The present study used a convergent parallel mixed-method study design (24) that integrated a cross-sectional quantitative survey and a qualitative analysis of interview responses (Figure 1). The specific objectives of this study were to a) assess general QOL among persons with SCI across sociodemographic and injury level factors (quantitative), b) explore which factors persons with SCI described as being associated with QOL in this population (qualitative) and c) integrate the quantitative and qualitative results to explain the comprehensive portrait of QOL of persons with SCI in Finland (mixed methods).

**Figure 1:**
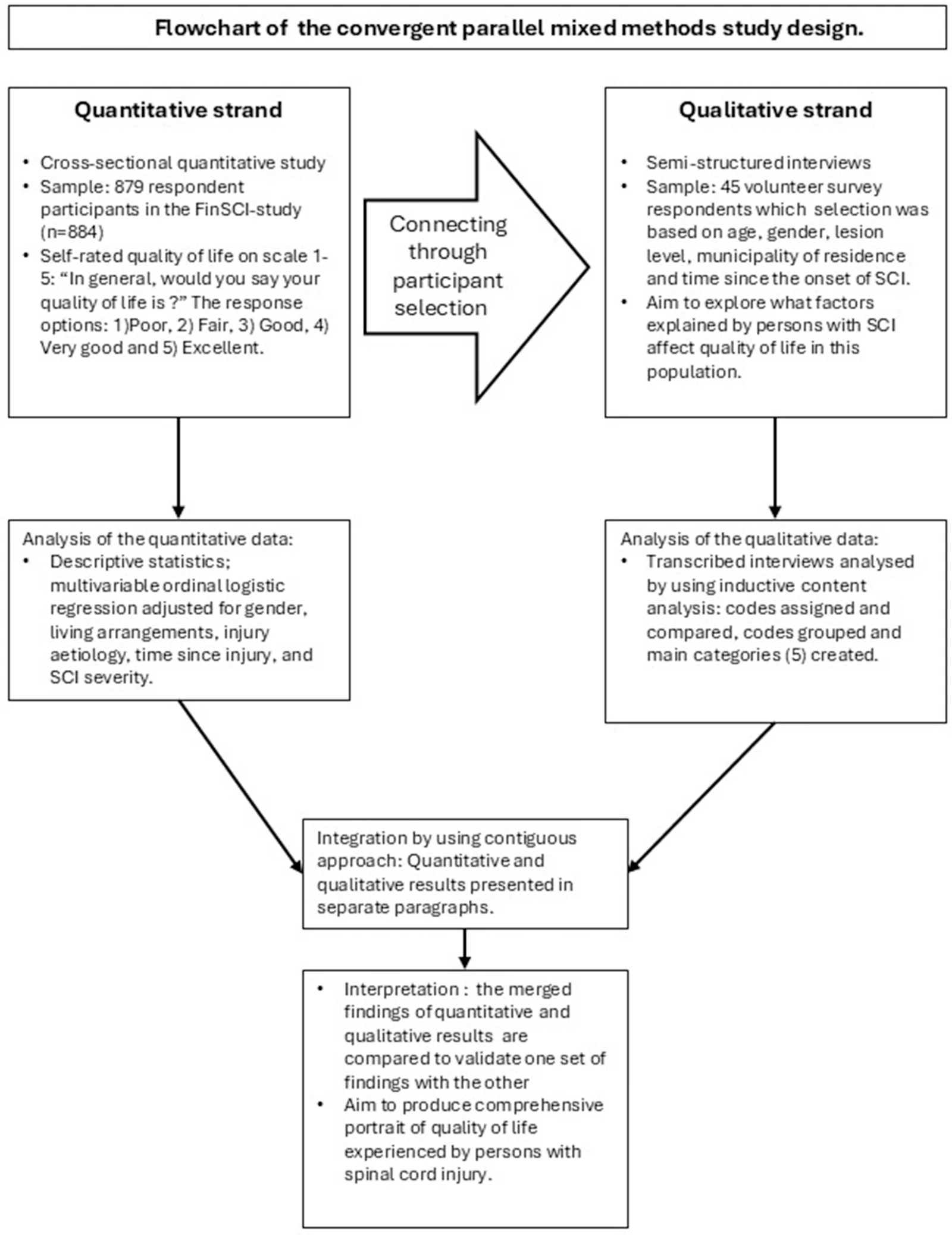
Flowchart of the convergent parallel mixed methods study design.

### 2.2 Data collection

Consistent with the logic of a parallel convergent mixed-methods design (24, 25), the quantitative and qualitative data was collected independently over a relatively short time lapse to answer the related research questions (25) and addressed with equal priority (24).

Data for the quantitative strand of the study was collected first. Quantitative data was collected and analyzed from 879 individuals with SCI who participated in the FinSCI (*n* = 884) and responded to the PROMIS® Global Health survey question: Global02 (26) concerning self-rated QOL on scale 1–5, socio-demographic and injury-related questions (23). The survey was implemented from February 2019 until the end of July 2019.

The qualitative phase was conducted from September to December 2019, during which data was collected through one-to-one semi-structured interviews from 45 volunteer survey respondents. The respondents for interviews were chosen by using a purposeful maximum variation sampling to ensure adequate representation of overall sample mirroring the distribution of the SCI population in Finland. The participants were systematically searched according to predetermined criteria: 5 participants for each of the three time periods since the injury: 1–5 years since SCI, 6–10 years since SCI, > 11 years since SCI. Also, age (20–30 years, 31–45 years, 46–60 years, 61–75 years, and > 75 years of age), gender, etiology (trauma or no trauma), and the severity of SCI were considered separately from the eligible population for three mentioned hospital regions covering the lifelong follow-up care of SCI patients in Finland. Fifteen participants were selected from each hospital district (Helsinki, Tampere, and Oulu) to ensure geographical representation and capture regional variation within the Finnish SCI population. Selection was based on a predetermined sampling matrix developed from the FinSCI registry data. Time since injury and age were the primary selection criteria, while gender, etiology, and SCI severity were considered to maximize variation within the sample. To enhance representation across SCI severity categories, the research group decided that each hospital district should include at least one participant from each SCI severity group.

During interview participant recruitment, it became clear that meeting the criteria was difficult due to the limited number of younger individuals with SCI in the FinSCI and their low willingness to participate (23). Specifically, 10 individuals declined the interview invitation, 5 of whom were under 30. As a result, more participants from the 31–45 age group were included. The final composition of participant characteristics in qualitative phase is shown in Table 1. Detailed characteristics of the interview participants (*n* = 45) are presented in Supplementary Table S1.

**Table 1:** Interview participants (*n* = 45).

Characteristic		n
Gender	Female	15
	Male	30
Age	20–30 years	6
	31–45 years	12
	46–60 years	10
	61–75 years	8
	>75 years	9
Time since injury	1–5 years	18
	6–10 years	14
	>11 years	13
Etiology	Trauma	28
	Non-trauma	17
Severity of SCI	C1–4 AIS A, B, C	5
	C5–8 AIS A, B, C	4
	T1-S5 AIS A, B, C	7
	AIS D	29

SCI = Spinal Cord Injury.

Groups C1–4 AIS A, B, C; C5–8 AIS A, B, C; T1-S5 AIS A, B, C and AIS D is the classification of severity of SCI based on the recommendations of The International Standards for Neurological Classification of SCI (ISNCSCI) (27).

Potential participants were contacted by phone to schedule interviews. The interviews were conducted in locations chosen by participants, mainly in their homes. Four took place in a quiet workspace arranged by the interviewers, and one in a public library meeting room. Most participants attended the interviews alone, though some were joined by their partners or adult children. A total of 22 participants were interviewed by the first author (KE) and 23 by the second author (ST). An average duration of interviews was 1 h and 10 min (range: 45 min to 2 h and 54 min).

The semi-structured interview template covered six themes, including QOL and well-being, rehabilitation, returning home and housing, functioning in daily life, employment, and accessibility. Interview questions were open to find out how respondents experienced QOL and factors affecting it. They were also asked to explain how their QOL has changed since the injury, how they saw their future, and whether they were able to influence the things that matter to them.

All participants were given written and verbal information about the study, and they gave their informed consent to participate in interviews. Participation in interviews was voluntary and could be terminated at any time. Participant confidentiality was assured. Data derived from the study was anonymized and stored electronically and physically in a research archive at the office of Finnish Association of People with Physical Disabilities.

### 2.3 Analysis

#### 2.3.1 Quantitative data procedures

The extent of missing data was assessed prior analyses. The response rate in the FinSCI survey was 49,9% (*n* = 884) among the eligible 1,772 participants. There were 879 respondents to the PROMIS® Global Health survey question: Global02 that measures QOL. Because proportion of missing data was very small (0.57%) and the sample size was large, we decided to employ listwise deletion method in SPSS to preserve the integrity and statistical rigor of our analyses by including only complete cases to analysis.

QOL was measured using the PROMIS® Global Health survey question (Global02), with response options of (1) Poor, (2) Fair, (3) Good, (4) Very good and (5) Excellent. Severity of SCI was reported and analyzed according to the International Spinal Cord Injury Core dataset (version 2.0) (27).

Associations between QOL and sociodemographic and injury-related variables were examined using multivariable ordinal logistic regression. All variables were entered simultaneously into the model to assess their independent associations with QOL.Results are presented as odds ratios (ORs), 95% confidence intervals (CIs), and *p*-values.

Statistical analyses were performed using IBM SPSS Statistics version 28.0 (28).

#### 2.3.2 Qualitative data procedures

All interviews yielded almost 53 total hours of data, from which 1,530 pages of text were generated. The interviews were tape-recorded and transcribed verbatim by professional typists with non-disclosure agreements, after which personal identifiers were removed and pseudonyms assigned by the interviewers (KE or ST). Subsequently, participants were pseudonymized by gender and are referred to using pseudonyms ranging from P1 to P45. The transcripts were combined into one file and transferred to the Atlas. Ti program (29) for coding. The aim of the qualitative data analysis was to inductively categorize participants' expressions on quality of life. The analysis followed Elo and Kyngäs (30) inductive content analysis. The research group comprised researchers and clinicians from social and health care disciplines, including four clinicians (KE, ST, JA, EK) with decades of experience in SCI care and three researchers (MZ, SH, MR). No prior relationship existed between the researchers and participants. Reflexivity was supported through regular group discussions to consider how researchers' backgrounds and assumptions might influence the analysis.

In preparation phase (KE) read transcriptions several times and chose themes for coding. The focus was on identifying the participants' expressions concerning factors associated with QOL. KE selected the most significant statements to represent the phenomenon, and citations (n574) were coded (n52). Coding and theme development emphasized relevance to the research questions and explanatory value rather than frequency alone. Following this condensation, the other authors (ST, MZ, JA, EK, MR, SH) individually reviewed the selected citations and codes. They participated in three common workshops to evaluate whether the codes suggested by (KE) effectively captured the essential elements representing the phenomenon. Any coding discrepancies or differences in interpretation were discussed collectively until consensus was reached on the final thematic structure. During this process, categories were refined to ensure conceptual distinctiveness, consistency in level of abstraction, and descriptive naming, while capturing the breadth of the data without forcing codes into categories. When needed, the team revisited the original data to verify interpretations. The categories were considered to provide a comprehensive representation of the data, and no further reorganization was deemed necessary.

The final number of codes was 51. After coding, the categories were grouped by (KE) to reduce the number of categories by collecting those that are similar into five high-order main categories. The five main categories and 51 subcategories are represented in Table 2. Descriptions of categories and their meanings are reported in the results section.

**Table 2:** Thematic categories of factors influencing positively or negatively on quality of life experienced by persons with spinal cord injury in Finland.

Category	Advancing	Either advancing or barring	Barring
Engaging in personally meaningful activities (9)	Ability to drive		Unable to do things used to do
	Able to do different things		Inability to give back
	Able to reciprocate		
	Caring for oneself		
	Continuity of lifestyle		
	Influencing one's own life		
	New meaningful doing		
Self-awareness and acceptance (14)	Acceptance of the situation	SCI as a turning point	Bad times
	Future dreams		Body deteriorates
	Satisfied with own functional capability		Dissatisfied with functional capability
	Personal growth		Limited life
	New values		Perceived strain
	Self-esteem		Reduced spontaneous activity and increased need for advance planning
	Work is meaningful		
Personal and societal attitudes (5)	Desire to be independent		Experience of unfair treatment
	Determination despite the disability		
	Live your life to the fullest		
	Treat me the same as before		
Coping with symptoms (6)			Bladder and bowel dysfunction
		Health status	Pain
		Mental well-being	Sexual dysfunction
			Spasticity
Social, physical and societal environment (17)	Assistive devices	Financial resources	Cold climate
	Basic needs met		Effect on relationships and social networks
	Care assistants		Inadequate health care services
	Family support		Inadequate social services
	Friends		Instructing care assistants is burdensome
	Help from close ones		Poor service process
	Help is well offered		Professionals' unawareness of the problem
	Peer support		
	Satisfied with housing		

#### 2.3.3 Mixed methods analysis

The independent quantitative and qualitative findings were integrated using a contiguous approach, with the results initially presented in separate sections (31) and subsequently compared to assess concordance, discordance or expansion during the interpretation phase (32). The analysis was guided by Moseholm and Fetters' (32) simultaneous bidirectional framework, in which both quantitative and qualitative findings serve as concurrent analytical lenses. Accordingly, the quantitative and qualitative results were examined iteratively in relation to one another, allowing both strands to contribute equally to the final interpretation and formation of overarching dimensions. Although the survey respondent pool was used as a sampling frame for the qualitative interviews and the interviews were conducted after the survey, this temporal sequence reflected a practical recruitment strategy rather than a sequential mixed-method design. Participants were selected according to predefined sampling criteria rather than quantitative findings, survey outcomes, or emerging results. Consequently, neither the design, data collection, nor analysis of the qualitative strand was informed by the quantitative strand. Instead, the qualitative component was conducted as an independent inquiry intended to provide a complementary perspective on the same phenomenon. Using overarching dimensions as an integrative framework, qualitative themes were compared with survey findings to identify areas of convergence or divergence. The integrated results were then presented in a joint display (Table 4) structured around the overarching themes. The findings of the mixed methods integration are reported in the Discussion section of this study.

## 3 Results

### 3.1 Quantitative results

Of the 884 respondents to the FinSCI survey, 879 individuals who answered the quality-of-life question were included in the study. Participants rated their overall QOL as fair (2), with a mean score of 2.6 (SD = 0.90) on a 5-point scale (Figure 2). Majority of survey respondents were men 574 (65.3%) and 305 (34.7%) were women. Their mean age was 61 (SD = 14, range = 20–90 years). Three participants were excluded from the multivariable analysis because of missing data in one or more model variables, resulting in a final analytical sample of 876 participants. The primary analysis consisted of an multivariable ordinal logistic regression model, which examined simultaneous associations of several participant characteristics on QOL.

**Figure 2:**
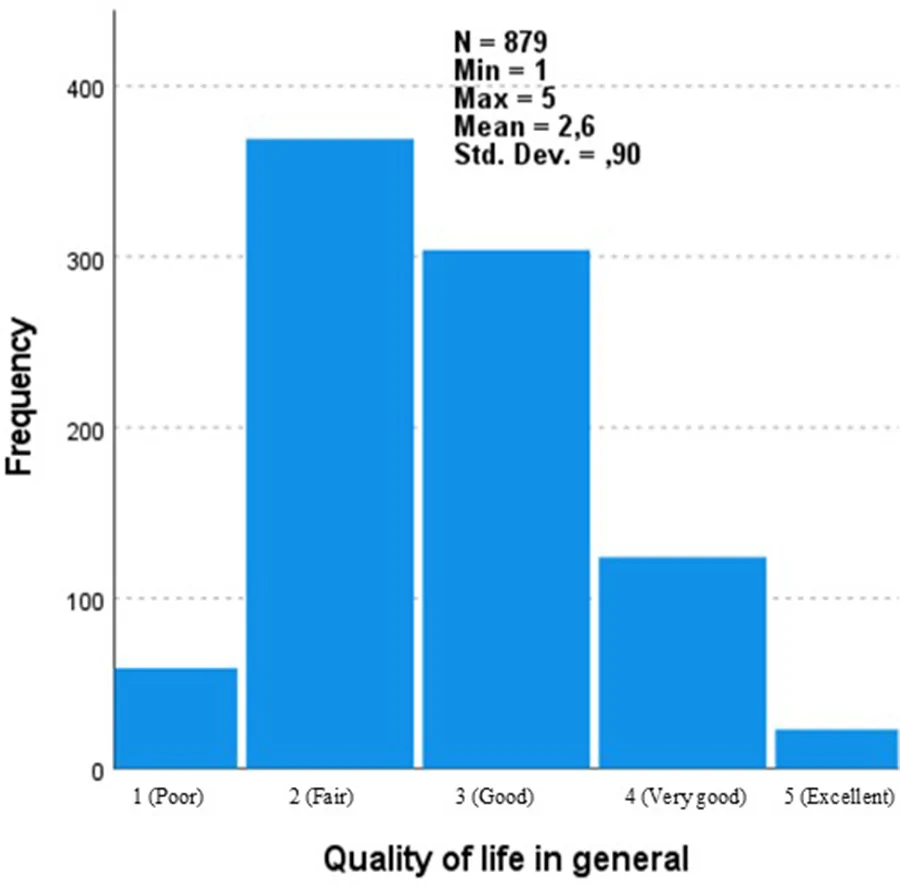
The quality of life in general in population of spinal cord injured persons in the Finnish Spinal Cord Injury Study (FinSCI, *n* = 879).

The multivariable ordinal logistic regression analysis was conducted to examine factors associated with QOL (PROMIS Global02). The model provided a significantly better fit to the data than the intercept-only model, as indicated by a reduction in the −2 log likelihood from 1039.74 to 963.44 [*χ*^2^(13) = 76.30, *p* < .001]. Model fit was evaluated using Pearson and Deviance goodness-of-fit statistics. The Pearson test was statistically significant, *χ*^2^(687) = 825.40, *p* < .001, whereas the Deviance test indicated an adequate fit, *χ*^2^(687) = 603.11, *p* = .990. Because the Deviance statistic is generally considered more reliable for assessing fit in ordinal logistic regression with large samples, these results suggest that the model fit the data adequately. The proportional odds assumption was assessed using the Test of Parallel Lines. The test was non-significant, *χ*^2^(39) = 43.33, *p* = .292, indicating that the parallel lines assumption was met and supporting the use of the ordinal logistic regression model.

Among the predictor variables, gender and age group were significantly associated with QOL (Table 3). Female participants had 1.38 times higher odds of reporting better QOL than male participants [OR = 1.38, 95% CI (1.05, 1.81), *p* = .021]. Compared with the oldest age group (≥76 years), all other age groups were significantly more likely to report higher QOL. The strongest association was observed for the youngest age group (16–30 years) [OR = 5.68, 95% CI (2.72, 11.88), *p* < .001], followed by age group 31–45 years [OR = 4.55, 95% CI (2.64, 7.85), *p* < .001], age group 46–60 years (OR = 2.00, 95% CI [1.28, 3.10], *p* = .002), and age group 61–75 years [OR = 1.91, 95% CI (1.28, 2.85), *p* = .002]. These findings indicate a gradient whereby younger age groups had progressively greater odds of reporting better QOL than the oldest age group.

**Table 3:** Results of the multivariable ordinal logistic regression analysis for quality of life (PROMIS Global02) in the Finnish spinal cord injury population (*n* = 876).

Variable	OR	95% CI	*p*-value
Gender			
Female	1.38	1.05–1.81	.021
Age group 16–30 years	5.68	2.72–11.88	<.001
Age group 31–45 years	4.55	2.64–7.85	<.001
Age group 46–60 years	2.00	1.28–3.10	.002
Age group 61–75 years	1.91	1.28–2.85	.002
Living arrangements			
Living alone	0.74	0.41–1.35	.330
In the household child/children	1.63	0.62–4.26	.324
With spouse, no children	1.09	0.60–1.97	.788
With spouse, in the household child/children	1.45	0.73–2.86	.285
Cause of the injury			
Traumatic	1.30	0.99–1.70	0.57
Severity of spinal cord injury			
C1–4 A, B, C	1.01	0.66–1.55	.959
C5–8 A, B, C	1.30	0.76–2.23	.332
T1-S5 A, B, C	1.09	0.79–1.52	.591

Reference categories: gender (male); age ≥76 years; living arrangements (another form of residence), cause of injury (NTSCI); severity of SCI (AIS D in all neurological levels).

OR, odds ratio; CI, confidence interval; SCI, spinal cord injury; NTSCI, non-traumatic spinal cord injury; AIS, American spinal injury association impairment scale.

Living arrangements were not significantly associated with QOL, as none of the comparisons with the reference category reached statistical significance (*p* values ranging from.285 to.788). Similarly, cause of injury was not a significant predictor, although the association approached significance [OR = 1.30, 95% CI (0.99, 1.70), *p* = .057]. The variable time since injury was initially included as a predictor in the multivariable ordinal logistic regression model but was effectively excluded from the final model because all associated parameters were identified as redundant by SPSS.

Finally, SCI severity was not significantly associated with QOL (all *p* > .05), indicating no evidence of differences in QOL across SCI severity categories after adjustment for the other variables in the model. Overall, the results suggest that age and gender were the primary factors associated with QOL, whereas living arrangements, cause of injury, and severity of SCI showed no significant independent associations in the adjusted ordinal logistic regression model (Table 3).

### 3.2 Qualitative results

In content analysis five categories were identified as being associated with quality of life of people with SCI: (1) Coping with symptoms, (2) Engaging in personally meaningful activities, (3) Personal and societal attitudes, (4) Self-awareness and acceptance and (5) Social, physical and societal environment. Each category was composed of multiple subcategories expressing barring or enhancing factors affecting QOL (Table 2).

#### 3.2.1 Coping with symptoms

The first category describes how health-related factors related to SCI affect a person's experience on QOL. Secondary health conditions (SHCs) such as pain, spasticity, bladder and bowel dysfunction, and sexual dysfunction were experienced as deteriorating factors to QOL (Table 2). Bowel and bladder problems were highlighted by interviewees under age 50 who actively engaged in daily life. Respondents described uncertainty about bowel and bladder function and extended time spent on toilet activities. Activities outside home were anticipated and people wanted to be sure that an accessible toilet was available due to uncertainty of bowel and bladder continence as well as need for regular catheterization.

Well, if we talk about bowel issues and catheterization, me and my wife have been discussing this and for me, this is the biggest issue in this situation, since it affects all that I do. So if I would need to choose, the first thing that I would take would be to be able to urinate and empty my bowel in a normal manner. After that I would take the ability to walk. So my bowel affects the most to where I can go and when and what I need to take with me. (P5)

Respondents identified the impact of mental health on QOL as either conducive or detrimental. They analytically described how mental well-being has varied either in terms of season or time since injury. Mental well-being was described as a balanced mental state in which the person is at peace with him/herself. On top of the injury, respondents identified factors that contributed to mental well-being, such as seasonal variation, pain, overall health status, work or unemployment.

Sexual dysfunction was described as negatively associated with intimate relationships, self-image and mental health. Men expressed erectile dysfunction and one female dyspareunia. Men experienced dissatisfaction with their sexual incapacity and assumed it also affected their relationship.

Of course, there was a little bit of those erection problems too, as nothing worked there, so it did make me really depressed. But then we started using the erection medication, and it worked. But since the sensory nerves were so damaged, I couldn't feel anything myself. It was completely numb. So, the erection worked with the help of medication, but there was no sensation, so I didn't get anything from it myself—except for the mental well-being that came from being able to make love to the person I was with. And that made up for it to a great extent. (P28)

Pain was reported to have the greatest disturbing impact on sleep, which in turn was related to daytime energy levels. Persistent pain over time also had a negative effect on mood, as pain levels often fluctuated, with participants experiencing both better and worse periods.

There is a huge variation in my days depending on how well I sleep and how much pain I'm in. Last night, for example, I was awake for about two hours in the middle of the night because my legs were simply so painful, and I couldn't find a comfortable position. I stayed awake until early morning so that I could take my morning medication. (P16)

#### 3.2.2 Engaging in personally meaningful activities

The interviewees expressed personally meaningful things for them that impacted their QOL despite the injury and it's effect on functioning. The responses showed that the respondents were engaging in activities that they liked and from which they found a sense of meaningfulness. After injury, returning to old activities or finding new interests suitable for oneself was found important.

The ability to function within the limits of one's functional capacity and to influence one's own life as desired were emphasized as factors promoting QOL. The interviewees expressed that they could impact personally meaningful matters by making choices that suited them and looking after themselves (appearance, health, fitness), noting that they were able to exercise control over their lives even when daily activities were carried out with assistance. What things are important to you? Well, it's that I can influence what my life is like and what I do. And then, that I can do things that make me feel good, and… that those things are made possible for me with additional help. (P1)

Acting as a peer support or participation in disability organization actions were presented as a meaningful activity to give fulfillment for oneself. Respondents wanted to participate in peer-to-peer activities and were disappointed if that was not possible. Several respondents would have liked to give back to the community or other disabled, but it was not always possible for different reasons such as lack of transportation or organization of activities. The aim of above-mentioned active participation was mentioned as a means to strengthen the coping of other people with disabilities or to share information for young people on road safety, for example.

I go to a peer support training organized by a spinal cord injury organization, and I want to do a peer support job. I can set the time in it for myself, and it doesn't bind me. If I can help colleagues with something, that's what I see as my future. (P12)

The change in physical ability after sustaining SCI forced some respondents to give up their previously active lifestyle or beloved hobbies, which were expressed as losses. Conversely, the opportunity to continue with previous hobbies or find alternative new activities was portrayed as having a positive impact on QOL. Access outside of home in different ways was expressed as a relevant factor for QOL. Driving a car independently increased the opportunities to decide one's own use of time and opportunities for mobility.

#### 3.2.3 Personal and societal attitudes

In this third category, respondents described how QOL can be affected by own internal or other's attitudes. How one reacts to and handles the harm caused by a disability is important to the QOL experience. The challenges or new actions were chosen to meet with perseverance and determined attitude. The highest possible level of independence in functioning was considered as an important goal. Despite the injury, there were expressions of positive and active attitudes towards life. Respondents shared their motto: “I make my life a full ten” (P32) or that “things don't get better by talking, they get better by doing” (P28).

Experiences of unfair treatment or discouraging attitudes of outsiders were related to negative decisions on healthcare services or social security benefits, or lack of understanding of the impairments caused by SCI. The respondents felt that they had not been heard or seen regarding their symptoms illustrating the far-reaching impact of SCI across multiple domains of life.

I have lost a sensation in my lower body at the age of 26, so anyone can imagine for themselves how that would feel… it's not just a quality-of-life issue. (P16).

The attitudes of those in the circle or families and friends towards the participants were described as encouraging, or neutral and expressions of treating in the same way as before were stated:

And no friend has ever abandoned me in any way or at all. In the beginning few guys were like they didn't really know how they would behave in that situation, I noticed that they were having a hard time. But they've also learned by now to realize that I am not different than before, I am the same guy but sitting in a wheelchair. (P8)

Although friends and family often required time to adapt to the new situation, positive adjustment was achieved when there was goodwill, willingness, and mutual understanding.

#### 3.2.4 Self-awareness and acceptance

After sustaining SCI, the respondents had reflected their self-image and identity. Experiencing a SCI was experienced as a turning point in their lives. Some younger respondents felt that SCI had brought a more mature attitude to their lives by forcing them to grow up earlier, while older respondents expressed that the SCI was linked to a decline in their QOL due to decreased functional ability.

Of course, quality of life comes from many things, but related to the topic, to stay close to it, my quality of life has certainly decreased significantly due to the decline in my physical ability. (P19)

Over time, the respondents' self-understanding of their abilities and disability had increased. They expressed greater self-compassion and an enhanced appreciation for everyday life. The respondents pronounced the acceptance of one's injury and the emergence of new values in their lives.

The respondents displayed satisfaction with their abilities and the actions enabled by these abilities. Working was perceived as containing an intrinsic value. Work was indicated as providing structure to the daily life, maintaining everyday routines, giving a sense of purpose, and fostering a feeling of being part of society.

For me, working is more about bringing routine and serving as a normal lifeline to everyday life. Financially, it's not very profitable, but it's a big mental thing as well—to have something to get up for and do. Working and having routines give me a reason to get up in the morning. (P4)

Although the respondents expressed considerable acceptance regarding the symptoms caused by SCI and their existing functional capacity, the impact of SCI on daily life was also experienced as limiting. The respondents described a decline in functional ability, which made daily tasks more time-consuming. Bad days and variation in days were understood as a natural part of life and were observed almost as from an outsider's perspective. In some situations, SCI was perceived as restrictive and narrowing one's social circle. Changes in functional capacity required greater effort to perform various activities and increased the need for advance planning, making spontaneous participation in everyday activities more difficult than for individuals without the condition.

Spontaneity is kind of excluded, you can't just do things like that. And if you want to do something, you have to prepare the situations, like, “Now we're doing this and that.” For example, if you plan to go somewhere farther, you have to plan it in advance for a longer time. You can't just decide to go somewhere for the weekend; it has to be planned ahead of time. (P4)

#### 3.2.5 Social, physical and societal environment

The social, physical, and societal environments significantly impact people's ability to function, as environmental factors can either promote or weaken it. Our study participants identified various environmental factors as either restricting or enhancing their ability to manage daily life and maintain well-being, thereby affecting their overall QOL.

The physical help and emotional support provided by people close to them was considered the most valuable and important issue in this study. Most of the respondents highlighted the importance of family members for good QOL.

On the other hand, in relationships, respondents spoke of the challenge of balancing care receiving with the dynamics of partnership. This balance either had a negative impact on the relationship or, when addressed, helped strengthen it. Those living alone found it especially challenging to form intimate relationships. Disability was also experienced to affect friendships—some were maintained despite the injury, while others were lost.

Respondents highlighted external factors that support their functional ability and daily life, such as assistive devices, an accessible living environment, and sufficient personal assistance. The value of peer support was also emphasized, particularly in providing emotional support and understanding of the situation. While personal assistants were viewed as helpful resources that ease everyday life, managing and directing them was seen burdensome. Respondents also expressed gratitude for having their basic daily needs met and for the stability in their lives.

And then there's the fact that you have a home. And that there's food… And also, that you're healthy, that there's nothing like… Even though you're in a wheelchair, you're still healthy in a way… Because it could be much worse. (P9)

Economic resources were portrayed as having either a positive or negative impact on QOL. The responses suggested that adequate financial resources enable a stable and sufficiently fulfilling life, while a tight financial situation forced individuals to make choices regarding everyday purchases and created pressure on the relationship.

The seasonal variation was highlighted as a factor affecting QOL, predominantly in a negative way. Winter, with cold weather and snow causing difficulties in moving with assistive devices and increasing stiffness in the body, reduced activities outside home.

Yes, it does get frustrating to some extent when you know the snow is coming again. Going outside decreases during that time. The cold is the worst, as you can't stay outside for very long in freezing temperatures.

The bureaucracy of social and healthcare services, as well as the insufficient services according to the respondents' experiences, were perceived as factors that negatively impacted QOL. The lack of knowledge or insufficient understanding among healthcare professionals about the specific factors related to SCI and SHCs was seen as a concern.

### 3.3 Mixed methods results

The quantitative and qualitative findings were largely complementary. Taken together, the findings suggest that factors beyond injury severity are important for understanding QOL after SCI. While injury severity was not independently associated with QOL in the adjusted quantitative model, the qualitative findings highlighted the importance of psychosocial, contextual, and adaptive factors. Qualitative findings helped explain the quantitative associations observed for age and the lack of an association between SCI severity and QOL. Some divergence was observed for gender, as female gender was associated with better QOL in the quantitative analysis, whereas no corresponding theme emerged from the qualitative data. Several additional factors associated with QOL, including SHCs and environmental factors, were identified only in the qualitative findings. The integrated findings and resulting overarching themes of mixed methods analysis are presented in Table 4 and discussed in detail in the Discussion section in relation to the existing literature.

**Table 4:** Mixed methods analysis of quality of life of persons with spinal cord injury in Finland.

Dimension	Quantitative findings	Qualitative findings	Mixed methods interpretation	Integration type
The impact of the severity of spinal cord injury on functioning and participation.	SCI severity was not independently associated with QOL^a^ (all *p* > .05).	The ability or inability of a person with a SCI* to participate in meaningful activities, maintain autonomy and influence one's own life affected perceived QOL.	The findings suggest that QOL is associated with autonomy, participation and engagement in personally meaningful activities following SCI. Injury severity itself was not independently associated with QOL.	Concordance + Expansion
The impact of age, health status or etiology on perceived QOL.	Younger age groups had significantly greater odds of reporting better QOL than the oldest age group (ORs 1.91–5.68, all *p* < .002). Females had higher odds of reporting better QOL than males (OR = 1.38, 95% CI, 1.05–1.81). Etiology was not independently associated with QOL (*p* = .057)	Secondary health conditions and overall health were perceived as important factors associated with QOL. No gender related theme emerged.	Age-related differences in QOL may be explained by health status, secondary health conditions, and their impact on everyday functioning and mental well-being. The quantitative finding regarding gender was not reflected in the qualitative data.	Concordance + Expansion; Discordance (gender)
Adaptation to spinal cord injury.	Time since injury could not be retained as an independent predictor in the final adjusted model.	Participants described increasing self-awareness, acceptance, adaptation, personal growth and changing life priorities.	The qualitative findings suggest that adaptation to SCI is a dynamic process involving personal growth and the development of strategies to manage everyday challenges, which may contribute to perceived QOL.	Expansion
The impact of social support and environmental factors on QOL.	Living arrangements were not independently associated with QOL (all *p* > .05).	Participants perceived family relationships, emotional and practical support, environmental accessibility, assistive devices, financial resources, climate and health and social services as factors associated with QOL.	Environmental factors, particularly social, were consistently described as facilitating or restricting participation, independence and well-being and were perceived as important factors associated with QOL.	Expansion

a QOL, quality of life.

* SCI, spinal cord injury.

## 4 Discussion

This study investigated the QOL of people with SCI in Finland and the factors that were associated with it using a mixed methods convergent design. Integrating quantitative and qualitative approaches provided a broader understanding of QOL and its determinants than either method alone. The quantitative and qualitative findings complemented each other. The quantitative data identified associations between participant characteristics and QOL while accounting for multiple variables simultaneously, providing population-level perspective on the phenomenon, whereas the qualitative data deepened the understanding of how individuals experienced and interpreted the factors affecting their QOL (see also 31). This integration represents a major strength of this study, as it enabled identification of both population level patterns and personal meanings attached to those experiences. In this study the QOL among people with SCI in Finland was rated numerically fair, but the qualitative responses revealed factors that enhance QOL, in addition to those that impair it. These factors are discussed below in the context of existing literature.

### 4.1 The impact of the severity of spinal cord injury on functioning and participation

This study produced results that align with a great deal of previous research in this field. Earlier studies have indicated that the level of injury itself does not appear to directly affect QOL; rather, its impact on QOL is reflected through limitations in functioning and participation (2, 7, 16, 17, 33–35). In the adjusted quantitative model, SCI severity was not independently associated with QOL. However, the qualitative findings demonstrated that the consequences of SCI on daily functioning remained highly relevant to participants' experiences. Respondents described reduced physical function, increased dependence on assistance, loss of spontaneity, and the need for greater planning in everyday life.

These findings complement previous analyses of the FinSCI dataset, showing that individuals in the AIS A, B, and C groups with higher neurological injury levels tend to have lower functional ability, whereas those with AIS D injuries, regardless of injury level, demonstrate the highest functional capacity (36). Nevertheless, the qualitative findings highlighted autonomy and perceived control over everyday life as important influences on participants' QOL, irrespective of injury severity. Similarly, Franceschini et al. (37) also identified that QOL is associated with a high level of autonomy. The qualitative narratives further suggested that engaging in personally meaningful activities within a social context—such as maintaining reciprocal relationships, finding new leisure activities, or having the capability to influence one's life, was perceived as important for QOL, as observed also in previous studies (19, 38–40). The integrated findings suggest that SCI severity influences QOL largely indirectly through its impact on functioning and participation. While the degree of neurological impairment affects functional capacity, autonomy and the ability to maintain control over everyday life appear to be closely associated with QOL.

### 4.2 The impact of age, health status or etiology on perceived QOL

Age was one of the strongest factors associated with QOL in the quantitative analysis, with younger age groups reporting significantly better QOL than older individuals. The qualitative findings were largely concordant with this result. Older respondents frequently described concerns related to declining physical functioning, maintaining health, and coping with age-related changes in addition to SCI, whereas younger participants more often discussed future-oriented goals, including employment, family formation, and active engagement in meaningful activities. Together, these findings suggest that age-related differences in QOL may be partly explained by differences in health status, functional capacity, and opportunities for participation.

Consistent with previous studies (41–43), older age combined with the physical limitations imposed by SCI may increase vulnerability to health-related challenges that negatively affect QOL. Individuals with NTSCI are generally older than those with traumatic injuries (44–46) and may therefore have a higher burden of SHCs and comorbidities (46, 47). In addition, the onset of NTSCI may be insidious, potentially resulting in delays in diagnosis and initiation of rehabilitation (44, 48). Although individuals with TSCI tended to report higher QOL than those with NTSCI, injury etiology was not independently associated with QOL in the adjusted model. This finding suggests that factors associated with aging and health status may be more important determinants of QOL than injury etiology itself. Similarly, Majamäki et al. (36) reported that SCI has a greater impact on health and functional ability than aging alone, contributing to declining independence over time.

The qualitative findings expanded the quantitative results by identifying SHCs, including pain, spasticity, sexual dysfunction, and bladder and bowel dysfunction, as prominent influences on QOL. Participants described how SHCs restricted everyday activities, reduced participation, and created uncertainty in social situations. These findings are consistent with previous research demonstrating that SHCs negatively affect QOL after SCI (2, 4–8). In particular, participants reported avoiding activities because of concerns about increasing pain, worsening spasticity, or bladder and bowel accidents, illustrating how SHCs may influence QOL indirectly through reduced participation and social engagement (1, 4). Previous studies have similarly shown negative associations between pain, spasticity, and QOL among individuals with SCI (8). Additional discussion regarding the impact of pain on work participation in this study population has been reported elsewhere by our research group (49). The quantitative and qualitative findings suggest that age-related differences in QOL are not explained by age alone. Rather, the integrated findings indicate that health status, SHCs, and their consequences for participation and everyday functioning may be important factors that may help explain association between increasing age and lower QOL. These results highlight a key mixed methods finding: while age emerged as an important predictor of QOL in the quantitative analysis, the qualitative data helped explain this association by revealing the central role of health-related challenges and SHCs in shaping everyday experiences and opportunities for participation. Consequently, rehabilitation services should prioritize the prevention and management of SHCs throughout the lifespan, as their effects extend beyond physical symptoms and may restrict participation in work, social relationships, and community life.

### 4.3 Adaptation to spinal cord injury

Previous studies have reported that QOL is positively associated with time since injury, suggesting that adaptation may occur over time following SCI (6, 33, 35, 42, 43, 50). However, conflicting findings have also been reported regarding the relationships between QOL, age, and time since injury (2, 33, 52). In the present study, the association between time since injury and QOL could not be formally evaluated in the adjusted quantitative model because SPSS identified all parameters related to time since injury as redundant.

The qualitative findings complemented these observations by providing insight into adaptation, acceptance, and personal growth may contribute to perceived QOL after SCI. Several participants described developing a greater appreciation of life, increased self-understanding, acceptance of their limitations, and a stronger sense of autonomy over time. These experiences are consistent with the concept of post-traumatic growth described by Tedeschi and Calhoun (51), referring to positive psychological changes that may emerge from struggling with a major life crisis, and align with previous findings among people living with SCI (11). The qualitative findings further suggested that adaptation involved more than adjusting to physical limitations. Participants emphasized the importance of maintaining control over everyday life, preserving autonomy, and continuing to engage in meaningful activities despite functional restrictions. These findings are consistent with previous studies identifying self-esteem, personal values, life perspective, autonomy, and perceived control as important contributors to QOL after SCI (1, 17). Respondents frequently described determination in facing challenges and a willingness to focus on remaining abilities rather than lost functions, illustrating how personal adaptation may buffer the impact of disability on QOL.

At the same time, the qualitative findings indicated that adaptation was neither uniform nor complete. Participants described losses in physical function, reduced spontaneity, increased dependence on planning, and restrictions in social participation as ongoing challenges affecting QOL. Some respondents expressed satisfaction with newly developed abilities and life perspectives, whereas others continued to experience sadness related to lost capabilities and opportunities. These findings suggest that adaptation may coexist with persistent functional limitations and their consequences for daily life. Consistent with previous studies (17, 56, 57), the loss of spontaneity was often portrayed as an accepted yet enduring aspect of living with SCI and requiring more effort and time compared to able-bodied persons.

Adaptation was also described as a process influenced by environmental and social circumstances. Previous research has suggested that employment may contribute positively to QOL among individuals aging with SCI (33, 54), whereas diminishing social support networks may negatively influence adjustment and well-being (43). These observations further support the view that adaptation to SCI reflects a dynamic interaction between personal resources and environmental factors. The quantitative and qualitative findings suggest that adaptation may be an important factor associated with QOL after SCI through which individuals maintain or improve QOL despite the long-term consequences of SCI. Although the adjusted quantitative model did not allow the independent effect of time since injury to be estimated, the qualitative findings offer a possible explanation for how acceptance, coping, autonomy, and personal growth may support perceived QOL over time. These findings support previous literature emphasizing that adaptation to SCI is a dynamic process shaped by both personal resources and environmental circumstances (43, 45, 53).

### 4.4 The impact of social support and environmental factors on QOL

Environmental and social factors emerged as important factors associated with QOL in the qualitative findings, complementing the quantitative results. Although living arrangements were not associated with QOL in the adjusted quantitative model, the qualitative data suggested that the quality and availability of social support may be more important than household composition itself. This finding highlights how the quantitative and qualitative data provided different but complementary perspectives on the role of social relationships in QOL after SCI.

According to the WHO's International Classification of Functioning, Disability, and Health (ICF), an individual's functioning reflects a dynamic and complex interaction between their health condition and contextual factors, including environmental elements (physical, social, and attitudinal) and personal factors (58). Consistent with previous SCI research (59, 60), the qualitative findings indicated that environmental factors could either facilitate or hinder participation and well-being. Unlike the studies by Bökel et al. (61) and Barclay et al. (62), in which the built environment was identified as a major barrier to participation and QOL, our participants more frequently emphasized the impact of cold climate and seasonal variation on daily activities, mobility, and social participation, in line with findings from other northern settings (63, 64).

The qualitative findings further expanded the quantitative results by identifying several environmental determinants of QOL that were not included in the regression model. Participants described inadequate healthcare and social services, limited professional knowledge regarding SCI-related complications, and burdensome service processes as negatively affecting their QOL, which is consistent with the findings of Gurung et al. (65). At the same time, participants expressed gratitude for the availability of assistance, as also reported by Boswell et al. (66), although coordinating and managing personal assistants was sometimes experienced as demanding. Together, these findings illustrate how environmental factors may be associated with QOL through their effects on autonomy, access to support, and participation in everyday life.

One of the strongest qualitative findings concerns the importance of social relationships. The participants reported that one of the most important factors associated with their QOL was close relationship with spouse and family members as reported also in studies by Kennedy and Rogers (67) and Jörgensen et al. (19). The support provided by family members was expressed both in a form of practical assistance with personal daily activities and emotional support. In previous literature, the strength of emotional relationships was expressed in two ways: the injury either strengthened or weakened interpersonal relationships (56). In our study some respondents analytically described changes in family relationships after SCI, similar to previous studies by Jeyathevan et al. (68) and Dickson et al. (69), in which individuals with SCI rely on their family members for support with activities of daily living (ADLs) and emotional support. Spinal cord injury can deteriorate the nature of intimate relationships by causing stress on the transformation of the relationship into a caregiving one. Opposite results regarding the significance of marriage on QOL have been expressed in studies by Gurcay et al. (70) and Celik et al. (15), in which culturally defined concept of family support can have an impact on this outcome.

Social support, meaning in general tangible or emotional support including peer support, has been found to have a positive effect on life satisfaction (67, 71) and promotion of participation (62) while loneliness has been reported to have a negative effect on the well-being of the disabled (72). Activities that individuals engaged in prior to their SCI are frequently altered significantly afterward, creating substantial challenges in preserving or re-establishing the relationships they previously had (55). The present findings suggest that social support was consistently described as an important factor associated with QOL not merely through the presence of other people, but through meaningful, reciprocal relationships that facilitate participation and a sense of belonging.

## 5 Limitations

This is the first study to provide data about QOL in general of people with SCI in Finland. The participants in this study were not representative of all people with SCI in Finland, but the attempt to obtain a representative sample of them was made through sampling. The number of young participants was low, so the lack of their opinion can bring bias to the results. The younger adults were underrepresented in the interview sample compared with the target sampling framework. This may have reduced the extent to which the findings captured experiences related to early adulthood, such as employment, intimate relationships, independence, and social participation.

Combining quantitative and qualitative data enriched the analysis but also introduced typical challenges of mixed methods research in interpreting the results, such as the challenge of integration. This was mainly due to the use of a single-item measure of general QOL (PROMIS® Global Health Global02), which provides a relatively narrow assessment of general QOL, whereas a broader range of validated instruments might have captured more diverse dimensions of the QOL construct.

Some findings may be particularly relevant to the Finnish context, including the influence of seasonal climate, which is not modifiable and may contribute to seasonal variation in participation and everyday activities. In contrast, findings related to autonomy, meaningful participation, psychosocial adaptation, social support, and SHCs are likely transferable beyond Finland, as they are consistent with the international SCI literature.

## 6 Conclusions

The integrated findings from this mixed-methods study suggest that psychosocial, health-related and contextual factors may play an important role in QOL after SCI, whereas injury severity was not associated with QOL in the adjusted quantitative analysis. The quantitative results revealed statistical relationships but provided a narrow analysis of the subject. In contrast, the qualitative results added context, depth, and personal reasoning regarding the factors affecting QOL. The novel contribution of this study lies in integrating population-level quantitative findings with qualitative accounts from persons living with SCI in Finland, providing a more comprehensive understanding of the factors associated with QOL after SCI.

Experiences of impaired overall health and poor management of SHCs were associated with reduced functional ability and poorer QOL, whereas SCI severity was not independently associated with QOL in the adjusted quantitative model. The study findings, taken as a whole, indicate that social support from loved ones, life experience—including psychosocial adaptation and personal growth over timewere perceived as important resources when coping with secondary health problems and functional limitations. Conversely, older age, secondary health problems, and inadequate health and social services were perceived as factors associated with poorer QOL.

To improve the QOL in this population, in addition to effective management of somatic symptoms, coping and adaptation should be supported, as these factors positively influence how individuals face recurring challenges. These findings underscore the need for comprehensive and person-centered rehabilitation strategies that extend beyond the management of physical impairments and SHCs and address the broader psychosocial dimensions of recovery. Interventions supporting long-term adaptation, social participation, autonomy, and access to appropriate health and social services may contribute to improved QOL among persons with SCI.

The participants in this study represented various levels of functioning and independence. Because the quantitative data were cross-sectional, the observed relationships should be interpreted as associations rather than evidence of causal or longitudinal effects. Although caution is warranted when generalizing the findings beyond the study population, the findings may help clinicians and rehabilitation professionals identify potentially modifiable factors associated with QOL and support the development of person-centered rehabilitation interventions that address not only physical functioning but also psychosocial, environmental, and participation-related factors associated with QOL.

## Data Availability

The datasets presented in this article are not readily available. The qualitative data used in this study cannot be shared due to participant confidentiality. The quantitative data may be made available upon reasonable request to the lead investigator (SH) of the Finnish Spinal Cord Injury Study (FinSCI). Requests to access the datasets should be directed to Sinikka Hiekkala, sinikka.hiekkala@invalidiliitto.fi
