# Supplementary material for: The quality of life experienced by persons with spinal cord injury in Finland – a mixed methods study
Source: Front Rehabil Sci. 2026 Oct 21;7:1829111. doi: 10.3389/fresc.2026.1829111 (PMC13731320; doi:10.3389/fresc.2026.1829111)
Supplement: Supplementary file 1 [file Table1.docx]

| Table 1. Interview participants in the Finnish Spinal Cord Injury Study (FinSCI), their general and lesion characteristics stratified by age and years since spinal cord injury (n=45). | | | |  |
| --- | --- | --- | --- | --- |
|  | 1–5 years since SCI | 6–10 years since SCI | >11 years since SCI |  |
| 20–30 years | P5, male, trauma 2017, T1-S5 AIS A, B, and C | P1, male, trauma 2010, C1-4 AIS A, B, C | P2, male, non-trauma 2008, C5-8 AIS A, B, and C |  |
|  | P6, female, trauma 2015, AIS D | P3, female, trauma 2010, T1-S5 AIS A, B, and C |  |  |
|  |  | P7, male, trauma 2012, AIS D |  |  |
| 31–45 years |  | P4, male, non-trauma 2011, T1-S5 AIS A, B, and C |  |  |
|  | P8, male, trauma 2015, C1-4 AIS A, B and C | P11, male, trauma 2014, C1-4 AIS A, B and C | P9, male, trauma 1995, C1-4 AIS A, B and C |  |
|  | P15, male, trauma 2016, AIS D | P12, male, trauma 2010, T1-S5 AIS A, B, and C | P10, male, trauma 2005, C1-4 AIS A, B and C |  |
|  | P18, male, trauma 2016, AIS D | P16, female, non-trauma 2012, AIS D | P13, male, trauma 1998, T1-S5 AIS A, B, and C |  |
|  |  | P17, female, trauma 2009, Group AIS D | P14, male, trauma 2008, AIS D |  |
| 46–60 years | P23, female, non-trauma 2015, AIS D | P25 male, non-trauma 2011, Group AIS D | P19, male, trauma 1979, C5-8 AIS A, B, and C |  |
|  | P24, male, non-trauma 2016, AIS D |  | P20, male, trauma 1993, C5-8 AIS A, B, and C |  |
|  | P26, male, trauma 2015, AIS D |  | P21, female, trauma 1983, T1-S5 AIS A, B, and C |  |
|  | P28, male, trauma 2018, AIS D |  | P22, male, trauma 2005, T1-S5 AIS A, B, and C |  |
|  |  |  | P27, male, trauma 1998, AIS D |  |
| 61–75 years | P29, female, non-trauma 2015, AIS D | P30, male, trauma 2011, AIS D | P32, female, non-trauma 2007, AIS D |  |
|  | P33, female, non-trauma 2016, AIS D | P31, female, non -trauma 2010, AIS D |  |  |
|  | P34, female, non-trauma 2015, AIS D | P35, male, non-trauma 2013, AIS D |  |  |
|  | P36, female, non-trauma 2015, AIS D |  |  |  |
| >75 years | P37, male, trauma 2016, C5-8 AIS A, B, and C | P41, male, non-trauma 2012, AIS D | P42, male, trauma 1999, AIS D |  |
|  | P38, male, trauma 2017, AIS D | P44, female, trauma 2011, AIS D | P43, female, non-trauma 2005, AIS D |  |
|  | P39, male, non-trauma 2014, AIS D |  |  |  |
|  | P40, female, non- trauma 2015, AIS D |  |  |  |
|  | P45, male, trauma 2016, AIS D |  |  |  |
| Note: SCI=spinal cord injury; Groups C1-4 AIS A, B, C; C5-8 AIS A, B, C; T1-S5 AIS A, B, C and AIS D is the classification of severity of SCI based on the recommendations of The International Standards for Neurological Classification of SCI (ISNCSCI) (27). | | | |  |
|  |  |  |  |  |
